# The Characteristic of Heat Wave Effects on Coronary Heart Disease Mortality in Beijing, China: A Time Series Study

**DOI:** 10.1371/journal.pone.0077321

**Published:** 2013-09-30

**Authors:** Zhaoxing Tian, Shanshan Li, Jinliang Zhang, Yuming Guo

**Affiliations:** 1 Emergency Department of Peking University Third Hospital, Beijing, China; 2 Department of Epidemiology and Biostatistics, School of Population Health, the University of Queensland, Brisbane, Australia; 3 State Key Laboratory of Environmental Criteria and Risk Assessment & Environmental Standards Institute, Chinese Research Academy of Environmental Sciences, Beijing, China; Univserity of Tolima, Colombia

## Abstract

**Background:**

There is limited evidence for the impacts of heat waves on coronary heart disease (CHD) mortality in Beijing, capital city of China.

**Objectives:**

We aimed to find a best heat wave definition for CHD mortality; and explore the characteristic of heat wave effects on CHD in Beijing, China.

**Methods:**

We obtained daily data on weather and CHD mortality in Beijing for years 2000–2011. A quasi-Poisson regression model was used to assess the short-term impact of heat waves on CHD mortality in hot season (May–September), while controlling for relative humidity, day of the week, long-term trend and season. We compared 18 heat wave definitions by combining heat wave thresholds (87.5^th^, 90.0^th^, 92.5^th^, 95^th^, 97.5^th^, and 99^th^ percentile of daily mean temperature) with different duration days (≥ 2 to ≥ 4 days), using Akaike information criterion for quasi-Poisson. We examined whether heat wave effects on CHD mortality were modified by heat wave duration and timing.

**Results:**

Heat wave definition using 97.5^th^ percentile of daily mean temperature (30.5 °C) and duration ≥ 2 days produced the best model fit. Based on this heat wave definition, we found that men and elderly were sensitive to the first heat waves of the season, while women and young were sensitive to the second heat waves. In general, the longer duration of heat waves increased the risks of CHD mortality more than shorter duration for elderly. The first two days of heat waves had the highest impact on CHD mortality. Women and elderly were at higher risks than men and young when exposed to heat waves, but the effect differences were not statistically significant.

**Conclusions:**

Heat waves had significant impact on CHD mortality. This finding may have implications for policy making towards protecting human health from heat waves.

## Introduction

Climate change is the biggest global health threat of the 21^st^ century and it affects directly or indirectly most populations [1]. Future climate change will increase both average and extreme temperatures [2-4]. There is strong evidence that heat waves have significant impacts on health [5]. For example, heat waves caused 15,000 excess deaths in the 2003 in France [6,7], and over 70,000 deaths across European countries [8,9].

Studies have examined the characteristic of heat wave effects on mortality [10,11]. For example, Anderson and Bell (2011) examined the modification of heat wave characteristics (e.g., intensity, duration, and timing in summer) on mortality in USA [10]. They reported that people were at higher risks when heat waves were more intense and longer, or occurred in early summer. Those studies on heat wave-related mortality have important implication for decision makers to design an early warning system, by establishing a heat wave threshold and the estimated increase in deaths above the threshold.

Elderly, children, and people with chronic diseases can be affected greatly by heat waves [12,13]. Coronary heart disease (CHD) is the second leading cause of cardiovascular death in the Chinese population, which accounts for 22% of cardiovascular deaths in urban areas and 13% in rural areas in China {Zhang, 2008 #56} [14]. There is clear evidence that the incidence of CHD is steadily increasing in China [15].

Many studies have reported that hot temperatures increase the risk of cardiovascular mortality and morbidity [16-19]. The heat wave events increased significantly during 1961–2007 in China, especially for northern China including Beijing [20]. However, there is little evidence on heat wave effects on CHD mortality in Beijing. To better protect people with coronary heart disease from heat waves, it is necessary and important to examine the characteristic of heat wave effects on coronary heart disease mortality in Beijing, China.

## Materials and Methods

### Data collection

Data on the daily counts of CHD deaths and climatic factors were collected from urban areas in Beijing, the capital of China. It is located in northern China, and has four distinct seasons, with hot, humid summers and cold, windy, dry winters.

This study only used daily counts of deaths, so ethic approval was not needed. Data on CHD deaths were collected from the Death Classification System, Beijing Public Security Bureau from January 2000 to December 2011. We classified CHD deaths according to the International Classification of Diseases, 10th revision (ICD-10: I20–I25). The CHD deaths were stratified by gender (women and men) and age (age ≥ 65 and age <65, using standard WHO definition) [21].

We collected climatic data on daily mean temperature and relative humidity from the China Meteorological Data Sharing Service System. The monitoring site of weather conditions is located at Daxing District (N39° 48' E116°28') in the southeast of Beijing. There was no missing data for temperature and relative humidity. We used only one monitoring site’s temperature to assess the heat wave effects on CHD mortality, because a previous study has shown that time series model using one monitoring site’s temperature is equal to spatiotemporal model using spatial temperatures for assessing city-wide temperature effects on mortality [22].

### Data analysis

#### Heat wave definition

There is no consistent heat wave definition worldwide, because people may acclimatise to their local climatic zones [23]. Generally, heat waves are defined by temperature threshold with consecutive days (duration) for a specific community. To compare which heat wave definition is the best to capture the effects on CHD mortality, we developed 18 heat wave definitions combining temperature thresholds (87.5^th^, 90.0^th^, 92.5^th^, 95^th^, 97.5^th^, and 99^th^ percentile of daily mean temperature) with duration of ≥2, ≥3, and ≥4 days.

We characterized each heat wave by its duration (length in days), and timing (first, second, or third heat wave of the season). Heat wave duration measured the heat wave’s length in days. We characterized the timing of the heat wave in the hot season by whether the heat wave was the first, second or third heat wave of its year. Also, we examined which heat wave days produced greater risk of CHD mortality.

#### Assessing heat wave effects

We examined the increase in CHD mortality during heat wave days compared with non-heat wave days in hot season (May–September), using a quasi-Poisson regression model which allows for the over-dispersion in daily counts of CHD deaths. Heat wave days were categorized as 1, while non-heat wave days were categorised as 0. We controlled for relative humidity using a distributed lag non-linear model with 5 degrees of freedom natural cubic spline for humidity and 4 degrees of freedom spline for lags up to 15 days. We controlled for day of the week as a categorical variable. We controlled for season using 4 degrees of freedom natural cubic spline for day of the year, and for long-term trend using a categorical variable for year. We examined model fit to assess which heat wave definition was the best by the sum of the Akaike information criterion for quasi-Poisson (Q-AIC) values from all group-specific CHD mortality.

Based on the best heat wave definition, we examined the characteristic of heat wave effects on CHD mortality (e.g., duration, timing, and which heat wave day produced higher risk). To examine whether longer duration of heat waves had greater impact on CHD mortality, we categorized the heat waves by their durations. Duration of 2 day is categorised as 2, 3 days=3, 4 days=4, etc. To examine whether the appearance timing of heat waves was a modifier for heat wave effects on CHD mortality, we used a categorical variable to identify heat wave timing. The first heat wave in each hot season was categorized as 1; the second was categorized as 2; the third was categorized as 3. To explore which day of heat waves had the higher risk of CHD mortality, we used a categorical variable to identify heat wave days. The first day of heat waves was categorized as 1, the second as 2, the third as 3.

Sensitivity analyses were performed by changing the degrees of freedom (3 to 6) and maximum lag from 10 to 30 days for relative humidity. We also changed degrees of freedom (5 to 10) for day of the year to control for seasonality. In addition, we examined whether the heat wave effects were driven by one or two special heat waves by repeating the analyses for each year.

All statistical tests were two-sided and *p*-values of less than 0.05 were considered statistically significant. R software (version 2.15.0) was used for all data analyses. “dlnm” package was used to fit distributed lag non-linear model for relative humidity [24].

## Results

Table 1 shows the sum of Q-AIC values from all group-specific CHD mortality for 18 heat wave definitions. Heat wave defined by daily mean temperature ≥ 30.5 °C (97.5th percentile) with duration ≥ 2 days gave the lowest Q-AIC value. Thus we used this heat wave definition in the following analysis to examine the characteristic of heat wave effects on CHD mortality.

**Table 1:** Value of the sum of the Akaike information criterion for quasi-Poisson (Q-AIC) values from all group-specific CHD mortality for each heat wave definition in hot season (May–September) in Beijing, China during 2000–2011.

Heat wave threshold	Values of Q-AIC		
(percentile of temperature)	Duration ≥ 2 days	Duration ≥ 3 days	Duration ≥ 4 days
87.5^th^ (28.3 °C)	31979.0	31970.3	31981.1
90.0^th^ (28.7 °C)	31976.6	31967.5	31981.8
92.5^th^ (29.1 °C)	31969.3	31972.6	31995.1
95.0^th^ (29.6 °C)	31972.6	31998.4	31994.2
97.5^th^ (30.5 °C)	**31959.8**	31998.9	32005.6
99.0^th^ (31.3 °C)	32019.3	32014.1	32019.2

Table 2 summarizes the daily weather and CHD deaths in hot season (May–September), heat wave days (daily mean temperature ≥ 30.5 °C with duration ≥ 2 days) in Beijing, China during 2000–2011. In general, heat wave days had higher counts of CHD death than non-heat wave days for all CHD groups.

**Table 2:** Mean (range) of daily weather and CHD deaths in hot season (May–September), heat wave days (temperature ≥ 97.5^th^ percentile of daily mean temperature with duration ≥ 2 days) in Beijing, China during 2000–2011.

	Hot season	Heat wave days	Non-heat wave days
Temperature (°C)	24.3 (11.2, 34.5)	31.5 (30.5, 34.5)	24.1 (11.2, 31.7)
Relative humidity (%)	61.2 (10, 95)	50.1 (21, 81)	61.4 (10, 95)
CHD deaths			
All	5 (0, 22)	7 (1, 11)	5 (0, 22)
Female	2 (0, 10)	2 (0, 6)	2 (0, 10)
Male	4 (0, 14)	5 (1, 9)	3 (0, 14)
Age < 65	2 (0, 12)	2 (0, 6)	1 (0, 12)
Age ≥ 65	4 (0, 14)	5 (1, 9)	4 (0, 14)

Table 3 shows the percentage increase of CHD mortality on heat wave days compared with non-heat wave days for 18 heat wave definitions in Beijing, China during 2000–2011. Generally, the higher heat wave threshold produced the higher risk for CHD mortality except for using 99th percentile. Heat wave definition using daily mean temperature ≥ 30.5 °C (97.5th percentile) with duration ≥ 2 days had the highest risk of CHD mortality. This is consistent with the model fit (Table 1). Generally, women were more sensitive to heat waves than men, while elderly were more vulnerable than young, but the differences were not statistically significant (results not shown).

**Table 3:** Percentage increase of coronary heart disease mortality on heat wave days compared with non-heat wave days for 18 heat wave definitions in hot season (May–September) in Beijing, China during 2000–2011.

Subgroup	Heat wave threshold	percentage increase (CIs)		
	(percentile of temperature)	Duration ≥ 2 days	Duration ≥ 3 days	Duration ≥ 4 days
All CHD	87.5^th^ (28.3 °C)	13.7 (5.7, 21.6)	16.5 (7.7, 25.3)	15.7 (6.1, 25.4)
	90.0^th^ (28.7 °C)	15.3 (6.4, 24.1)	18.3 (8.5, 28.1)	18.5 (7.1, 29.9)
	92.5^th^ (29.1 °C)	18.1 (8.4, 27.8)	18.3 (7.9, 28.7)	16.2 (3.8, 28.7)
	95.0^th^ (29.6 °C)	20.7 (9.5, 32.0)	16.4 (3.2, 29.6)	20.2 (5.2, 35.2)
	97.5^th^ (30.5 °C)	31.0 (16.5, 45.6)	23.2 (5.2, 41.2)	21.0 (0.3, 41.7)
	99.0^th^ (31.3 °C)	10.2 (-20.5, 40.9)	22.9 (-9.6, 55.4)	12.6 (-32.6, 57.7)
Female	87.5^th^ (28.3 °C)	16.0 (2.5, 29.5)	21.8 (6.9, 36.6)	26.7 (10.7, 42.7)
	90.0^th^ (28.7 °C)	15.4 (0.3, 30.5)	21.9 (5.4, 38.4)	24.3 (5.1, 43.4)
	92.5^th^ (29.1 °C)	18.5 (2.0, 35.0)	24.1 (6.7, 41.5)	25.2 (4.5, 45.8)
	95.0^th^ (29.6 °C)	20.7 (1.4, 40.0)	18.0 (-4.6, 40.7)	32.8 (7.9, 57.7)
	97.5^th^ (30.5 °C)	37.3 (12.7, 61.8)	30.9 (0.5, 61.3)	28.1 (-7.1, 63.4)
	99.0^th^ (31.3 °C)	25.7 (-22.0, 73.4)	39.3 (-10.8, 89.3)	45.9 (-18.7, 110.6)
Male	87.5^th^ (28.3 °C)	12.6 (3.2, 21.9)	14.0 (3.6, 24.4)	10.4 (-1.1, 21.9)
	90.0^th^ (28.7 °C)	15.2 (4.8, 25.6)	16.6 (5.0, 28.1)	15.7 (2.1, 29.2)
	92.5^th^ (29.1 °C)	17.8 (6.4, 29.3)	15.5 (3.2, 27.7)	11.9 (-2.9, 26.7)
	95.0^th^ (29.6 °C)	20.7 (7.5, 33.9)	15.6 (0.1, 31.1)	14.1 (-3.8, 32.0)
	97.5^th^ (30.5 °C)	28.0 (10.8, 45.3)	19.5 (-1.8, 40.8)	17.6 (-6.7, 42.0)
	99.0^th^ (31.3 °C)	1.5 (-36.5, 39.5)	13.5 (-26.9, 53.9)	-8.8 (-68.1, 50.5)
Age<65	87.5^th^ (28.3 °C)	11.8 (2.7, 20.8)	13.8 (3.7, 23.9)	14.8 (3.8, 25.7)
	90.0^th^ (28.7 °C)	10.8 (0.7, 21)	12.2 (0.9, 23.4)	13.0 (-0.2, 26.1)
	92.5^th^ (29.1 °C)	12.2 (1.0, 23.4)	11.3 (-0.7, 23.3)	11.5 (-2.8, 25.8)
	95.0^th^ (29.6 °C)	15.9 (2.9, 28.9)	11.1 (-4.2, 26.3)	15.2 (-2.2, 32.6)
	97.5^th^ (30.5 °C)	25.9 (9.1, 42.8)	26.6 (6.5, 46.8)	29.0 (6.3, 51.7)
	99.0^th^ (31.3 °C)	16.0 (-18.1, 50.1)	29.5 (-6.4, 65.3)	15.1 (-35.3, 65.6)
Age≥65	87.5^th^ (28.3 °C)	18.4 (3.7, 33.1)	23.2 (7.0, 39.5)	18.1 (0.1, 36.2)
	90.0^th^ (28.7 °C)	26.4 (10.2, 42.5)	33.4 (15.7, 51.1)	32.2 (11.5, 52.8)
	92.5^th^ (29.1 °C)	32.6 (15.0, 50.2)	35.4 (16.8, 54)	28.3 (5.7, 50.9)
	95.0^th^ (29.6 °C)	32.6 (12.2, 52.9)	29.4 (5.7, 53.0)	32.5 (5.4, 59.5)
	97.5^th^ (30.5 °C)	43.4 (17.2, 69.6)	13.4 (-21.8, 48.6)	-3.5 (-46.8, 39.7)
	99.0^th^ (31.3 °C)	-6.2 (-67.5, 55.2)	3.6 (-62.9, 70.1)	5.5 (-83.3, 94.3)

**Figure 1:**
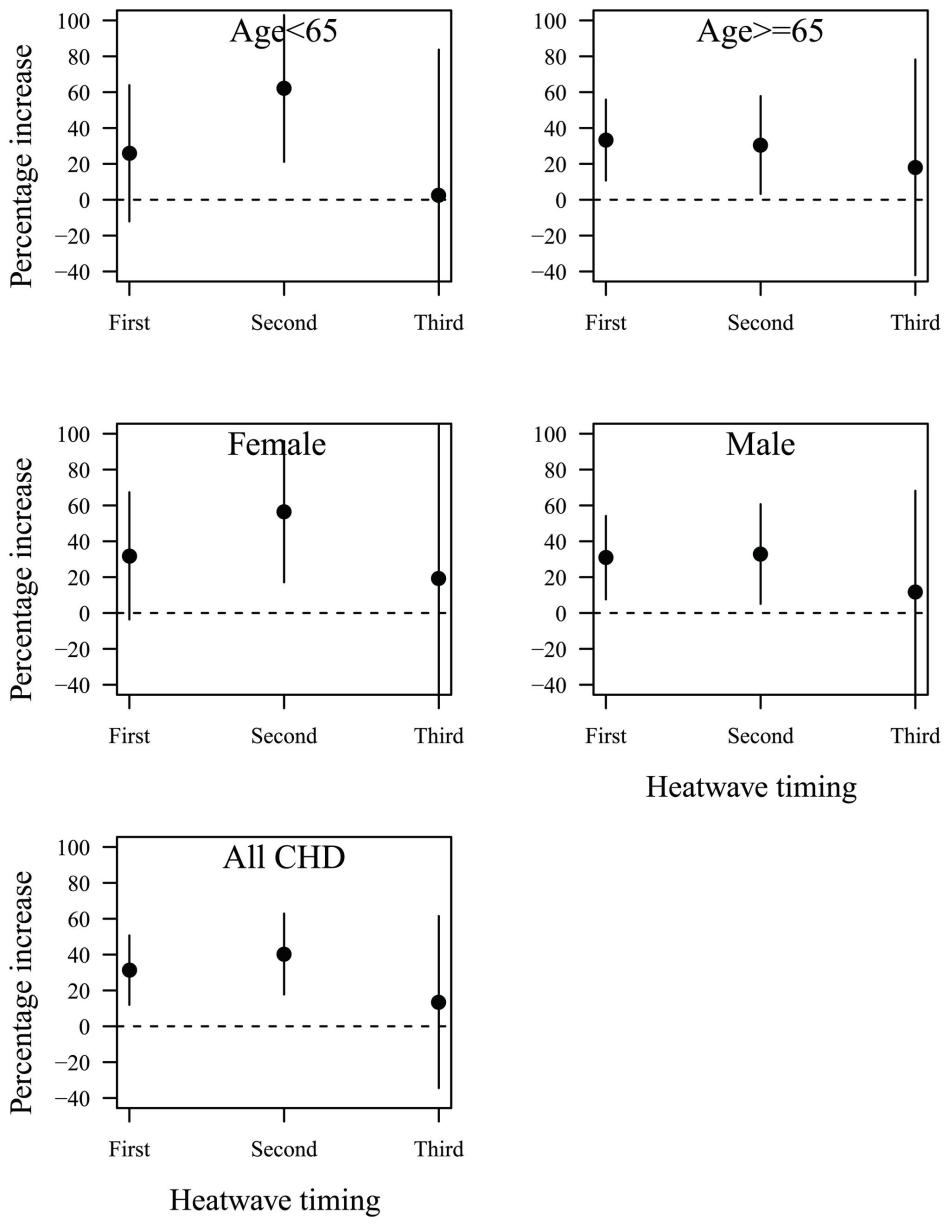
Heat wave timing and heat wave effects on CHD mortality in hot season (May–September) in Beijing during 2000–2011: percentage increase of CHD mortality on heat wave days compared with non-heat wave days; heat wave was defined by daily mean temperature ≥ 97.5^th^ percentile with duration ≥ 2 days.

Figure 1 shows the heat wave timing and heat wave effects on CHD mortality. Old and men were sensitive to the first heat waves, while the young and women were sensitive to the second heat waves of the season.

**Figure 2:**
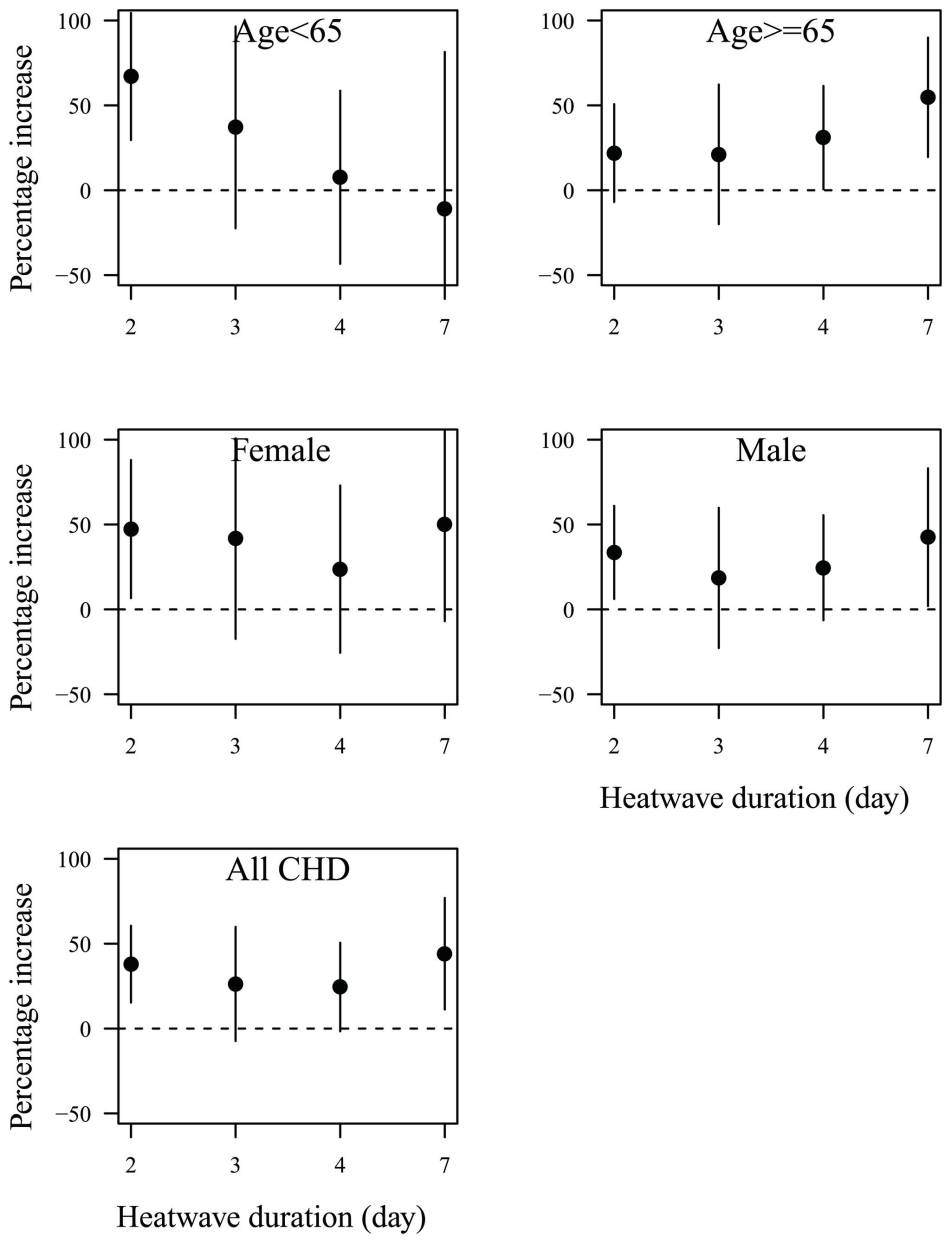
Heat wave duration and heat wave effects on CHD mortality in hot season (May–September) in Beijing during 2000–2011: percentage increase of CHD mortality on heat wave days compared with non-heat wave days; heat wave was defined by daily mean temperature ≥ 97.5^th^ percentile with duration ≥ 2 days.

Figure 2 shows the heat wave duration and heat wave effects on CHD mortality. The longer duration of heat waves increased the risks of CHD mortality more than shorter duration among the elderly, while short duration increased the risk among the young, women, and men.

**Figure 3:**
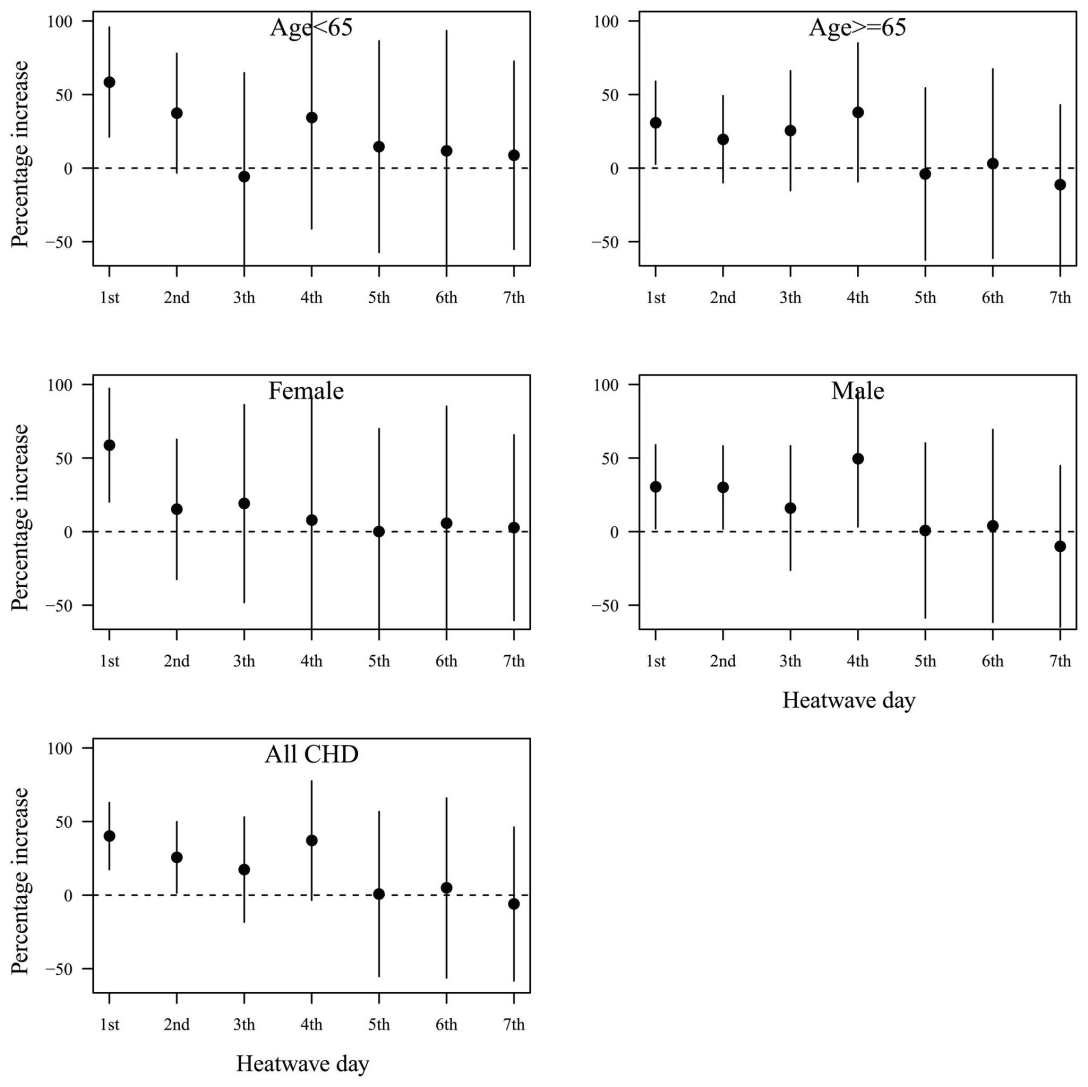
The day of heat waves and heat wave effects on CHD mortality in hot season (May–September) in Beijing during 2000–2011: percentage increase of CHD mortality on heat wave days compared with non-heat wave days; heat wave was defined by daily mean temperature ≥ 97.5^th^ percentile with duration ≥ 2 days.

Figure 3 shows the day of heat wave and heat wave effects on CHD mortality. In general, only the first two days of heat waves significantly increased the risk of CHD mortality.

The change of lag from 10 to 30 days, and the degrees of freedom (3 to 6) for relative humidity did not substantially change the effect estimates. Time series models using degrees of freedom (5 to 10) of the year produced similar results as our main findings. Our results show that all heat waves had similar adverse effects on mortality (results not shown). To check our main findings, we conducted a time-stratified case-crossover analysis using calendar month as strata. The results are similar as our findings (results not shown).

## Discussion

To our knowledge, this is the first study to examine heat wave effects on CHD mortality in China. In this study, we found that heat wave definition using daily mean temperature ≥ 30.5 °C (97.5th percentile) with duration ≥ 2 days is the best to capture heat wave effects on CHD mortality in Beijing. Heat waves significantly increased the risks of CHD mortality for all groups. Women and elderly were more sensitive than men and young, respectively, even though these differences were not statistically significant. The characteristic of heat wave effects on CHD mortality differed by gender and age. We also found that only the first two days of heat waves had significant effects on CHD mortality.

It is hard to find a global heat wave definition [23]. Generally, there are two types of heat wave definitions: one is that using temperatures above a threshold that is physiologically based (absolute threshold) with consecutive days; the other one is that using location based (relative threshold) with consecutive days [25]. In this study we used a relative threshold based heat wave definition, which considers local long-term weather and allows for acclimatization to local weather types. We compared 18 heat wave definitions and found that using daily mean temperature ≥ 30.5 °C (97.5th percentile) with duration ≥ 2 days is the best to capture heat wave effects on CHD mortality. The heat wave definition with duration>=2 days includes all heat waves that lasted for more than 2 days, but the heat wave definition with duration>=4 days did not include those heat waves with duration of 2-3 days. These mean that the heat wave definition with duration>=2 days can fully capture the impacts of heat waves on CHD mortality, but the heat wave definition with longer duration cannot. This finding is important and useful for local government to make efficient policy for protecting people from extreme temperatures.

It is hard to compare our results with previous studies, because very few studies have specially focused on heat wave effects on CHD mortality. However, many studies have reported that heat waves have adverse impacts on non-accidental and cardiovascular mortality and morbidity [26,27]. For example, in Moscow, excess non-accidental mortality, coronary heart disease mortality, cerebrovascular mortality during the 2001 heat wave were 33% (95% CI: 20%, 46%), 32% (95% CI: 16%, 48%), and 51% (95% CI: 29%, 73%), respectively [28]. In Brisbane, the number of non-accidental deaths and emergency hospital admissions during heat waves increased by 62% (95% CI: 36%, 94%) and 22% (95% CI: 14%, 30%) in comparison with non-heat wave days [29].

It seems that elderly and women were more sensitive to heat waves than young and men, but the estimate differences were not statistically significant. This is consistent with previous studies [30]. The reason might be that the thermal regulation system weakens with aging, for example, skin sensory perception may diminish and thermal homeostasis may decline [31]. Women have higher risks for ischemic, arrhythmic and blood pressure which are associated with the heat waves [32].

In this study, we found that elderly and men were sensitive to first heat waves, while young and women were sensitive to second heat waves. Previous studies have reported that heat wave timing may modify the heat wave effects on mortality [10]. Early appeared heat waves had higher mortality risk than late appeared heat waves. This might be because first heat waves killed most of susceptible population (e.g., elderly) during the hot season, and a smaller pool of susceptible individuals was left (mortality displacement) or because *people have ability to* acclimatize to hot weather over the course of hot season [33,34]. Further detailed studies are needed to determine why men were sensitive to early heat waves while women sensitive to later heat waves, for example, behaviour modification and biological adaptation.

Previous studies provided evidence that of longer heat wave duration had greater mortality risk [10,35]. However, we found that the effects of heat wave duration on CHD mortality differed by age and gender. The longer duration had higher risks for the elderly, while short duration produced higher risks for young, women and men. The reason might be that they have different activity, behaviour and biological adaptation.

To better protect people from heat waves and reasonably use public resources, it is important to understand which day of heat waves increases the risk of mortality [36]. We found that only the first two days of heat waves increased the risk of CHD mortality. Previous studies have reported that the effects of heat waves appeared within a short time frame [37,38]. For example, the 2003 French heat wave quickly increased the risk of mortality in most cities during a 4 days’ period [7]. Similar results were reported by the Chicago heat wave study [39]. Previous studies have shown that heat waves have immediate effects on people with cardiovascular diseases [40,41]. Those findings suggest that cardiovascular deaths (including CHD) might happen quickly when they become exposed to heat waves, as their ability to cope with heat is already compromised.

Our findings are useful for developing warning systems. Public health workers should take the action to educate communities on the health risks of heat waves, and teach them how important it is to reduce exposure to the heat and to increase the access to the cool environment. Policy makers should implement an early warning system to prevent heat wave effects on human health including early alerts and advisories, as well as implement a variety of emergency measures to mitigate the heat dangers [42]. Meanwhile, it is very important to add staff and increase their rotation for hospitals, clinics, and health care during heat waves.

There are also some limitations in this study. We only focused on one city, so the results might not be generalised to other areas. We did not control for air pollution, as these data were not available. However, some studies found that the temperature effects on mortality are robust after controlling for air pollution [43]. Future studies are still needed to look at this issue.

## Conclusions

The findings of this study demonstrate that heat waves had significant impacts on CHD mortality. We suggest using daily mean temperature ≥ 30.5 °C (97.5th percentile) with duration ≥ 2 days as heat wave definition in Beijing, as it was the best to capture heat wave effects on CHD mortality, and as only the first two days of heat waves had significant effects on CHD mortality. The characteristics of heat wave effects on CHD mortality differed by gender and age group. Our findings are important to develop efficient heat warning system in Beijing, China.
